# Higher airborne pollen concentrations correlated with increased SARS-CoV-2 infection rates, as evidenced from 31 countries across the globe

**DOI:** 10.1073/pnas.2019034118

**Published:** 2021-03-08

**Authors:** Athanasios Damialis, Stefanie Gilles, Mikhail Sofiev, Viktoria Sofieva, Franziska Kolek, Daniela Bayr, Maria P. Plaza, Vivien Leier-Wirtz, Sigrid Kaschuba, Lewis H. Ziska, Leonard Bielory, László Makra, Maria del Mar Trigo, COVID-19/POLLEN study group, Claudia Traidl-Hoffmann

**Affiliations:** ^a^Chair of Environmental Medicine, Technical University of Munich, Augsburg 86156, Germany;; ^b^Institute of Environmental Medicine, Helmholtz Centre Munich, Augsburg 86156, Germany;; ^c^Department of Environmental Medicine, Faculty of Medicine, University of Augsburg, Augsburg 86156, Germany;; ^d^Finnish Meteorological Institute, Helsinki FI-00101, Finland;; ^e^Mailman School of Public Health, Columbia University, New York, NY 10032;; ^f^Center for Environmental Prediction, Rutgers University, New Brunswick, NJ 08901;; ^g^Environmental and Occupational Health Science Institute, Rutgers University, Piscataway, NJ 08854;; ^h^Medicine, Allergy, Immunology and Ophthalmology Department, Hackensack Meridian School of Medicine, Nutley, NJ 07110;; ^i^New Jersey Center of Science, Technology and Mathematics, Kean University, Union, NJ 07083; ^j^Institute of Economics and Rural Development, Faculty of Agriculture, University of Szeged, Szeged 6720, Hungary;; ^k^Department of Botany and Plant Physiology, University of Malaga, Malaga 29016, Spain

**Keywords:** COVID-19, pollen, viral infection, aerobiology

## Abstract

Pollen exposure weakens the immunity against certain seasonal respiratory viruses by diminishing the antiviral interferon response. Here we investigate whether the same applies to the pandemic severe acute respiratory syndrome coronavirus 2 (SARS-CoV-2), which is sensitive to antiviral interferons, if infection waves coincide with high airborne pollen concentrations. Our original hypothesis was that more airborne pollen would lead to increases in infection rates. To examine this, we performed a cross-sectional and longitudinal data analysis on SARS-CoV-2 infection, airborne pollen, and meteorological factors. Our dataset is the most comprehensive, largest possible worldwide from 130 stations, across 31 countries and five continents. To explicitly investigate the effects of social contact, we additionally considered population density of each study area, as well as lockdown effects, in all possible combinations: without any lockdown, with mixed lockdown−no lockdown regime, and under complete lockdown. We found that airborne pollen, sometimes in synergy with humidity and temperature, explained, on average, 44% of the infection rate variability. Infection rates increased after higher pollen concentrations most frequently during the four previous days. Without lockdown, an increase of pollen abundance by 100 pollen/m^3^ resulted in a 4% average increase of infection rates. Lockdown halved infection rates under similar pollen concentrations. As there can be no preventive measures against airborne pollen exposure, we suggest wide dissemination of pollen−virus coexposure dire effect information to encourage high-risk individuals to wear particle filter masks during high springtime pollen concentrations.

## Significance

Coexposure to airborne pollen enhances susceptibility to respiratory viral infections, regardless of the allergy status. We hypothesized this could be also true for SARS-CoV-2 infections. To investigate this, we tested for relationships between SARS-CoV-2 infection rates and pollen concentrations, along with humidity, temperature, population density, and lockdown effects. Our unique dataset derives from 130 sites in 31 countries and across five continents. We found that pollen, sometimes in synergy with humidity and temperature, explained, on average, 44% of the infection rate variability. Lockdown halved infection rates under similar pollen concentrations. As we cannot completely avoid pollen exposure, we suggest wide dissemination of pollen−virus coexposure information to encourage high-risk individuals to wear particle filter masks during high springtime pollen concentrations.

Progress of COVID-19 is presumed to be often asymptomatic or associated with only mild to moderate symptoms, mainly fever and dry cough (1). However, in susceptible individuals, such as elderly persons with metabolic, cardiovascular, and/or pulmonary comorbidities (2), COVID-19 can exacerbate to severe pneumonia requiring oxygen supplementation and intensive care treatment. COVID-19−associated deaths are mainly due to severe acute respiratory syndrome (SARS), cytokine storm (3–5), or disseminated coagulopathy leading to multiorgan failure. According to World Health Organization (WHO) estimates, the overall case fatality rate is 3.4% (6, 7).

SARS coronavirus 2 (SARS-CoV-2), the causative of COVID-19, is a novel member of the Betacoronaviridae family with presumed zoonotic origin (8). It is a positive-stranded RNA virus with a genome size of ∼30 kb (9). SARS-CoV, the agent of the SARS epidemic of 2002 and its closest related sibling, is highly susceptible to antiviral interferons (IFNs) and has developed immune suppression mechanisms on the basis of antagonizing host cell IFNs. The accessory proteins encoded by the genes ORF3b, ORF6, M, and N of SARS-CoV-2 are highly homologous to their SARS-CoV and Middle East respiratory syndrome counterparts, which are type I IFN antagonists (10). Another set of accessory proteins, encoded by the genes E, ORF3a, and ORF8b and common to both SARS-CoV and SARS-CoV-2, are activators of the NLRP3 inflammasome (11, 12) and have up to 95% interstrain amino acid sequence identity (9). Excessive inflammasome activation and subsequent pyroptosis is the underlying mechanism for the IL-1β dominated cytokine storm associated with SARS-CoV mediated multiorgan failure (4, 13).

A recent, large cohort study from South Korea reported that asthma exacerbations in school-aged children are associated with coexposure to multiple seasonal environmental factors, that is, ozone, rhinovirus, and tree pollen (14). Another study recently reported that pollen grains of various plant taxa release as yet unidentified compounds that down-modulate the production of antiviral λ-IFNs in respiratory epithelial cells, and provided evidence from human and mouse models that pollen exposure leads to enhanced susceptibility to infection with two different respiratory viruses, human rhinovirus and respiratory syncytial virus (15). Also, some pollen types enhance the release of the IL-1 family cytokines IL-1β, IL-18, and IL-33 from epithelial cells in vitro, indicating a role for pollen in NLRP3 inflammasome activation (16, 17). Thus, two mechanisms of the innate immune response, inflammasome activation and antiviral IFN response, appear to be modulated toward the same direction by pollen and SARS-CoV-2.

The first COVID-19 cases were officially reported for European countries at the middle to end of January 2020. On 12 March, the WHO officially announced the onset of a global COVID-19 pandemic, with over 33% of the world´s nations reporting local spreading of the infection. Around the same time, a large-scale warm spell across the bulk of the Northern Hemisphere initiated the first large seasonal peak in tree pollen emissions. The synchronized timing of the spreading of the infection and the higher pollen concentrations, in combination with the recently found potential of pollen to enhance susceptibility for respiratory viruses, prompted us to analyze whether, under certain weather conditions, a positive correlation between SARS-CoV-2 infections and airborne pollen could be observed. We therefore collected airborne pollen data from most pollen monitoring stations operating at that time, from a total of 31 countries and from all inhabited continents, including both the Northern and Southern Hemispheres, and investigated for relationships between daily pollen concentrations and SARS-CoV-2 infection rates, also taking meteorological and sociodemographic factors into account.

Our results reveal that the simultaneous exposure to SARS-CoV-2 (via other infected human carriers) and airborne pollen may, under “favorable” weather conditions, promote viral infection. While it is meaningful to inform the public about this risk, the wording should be extremely well considered to avoid misunderstandings and to not cause panic. On the other hand, wide dissemination of the potential dire effects of virus−pollen coexposure ought to be urgently and clearly communicated: As we cannot avoid airborne pollen exposure, high-risk groups have to be informed to wear particle filter masks during the pollen season, especially in springtime.

## Results

**Fig. 1.**
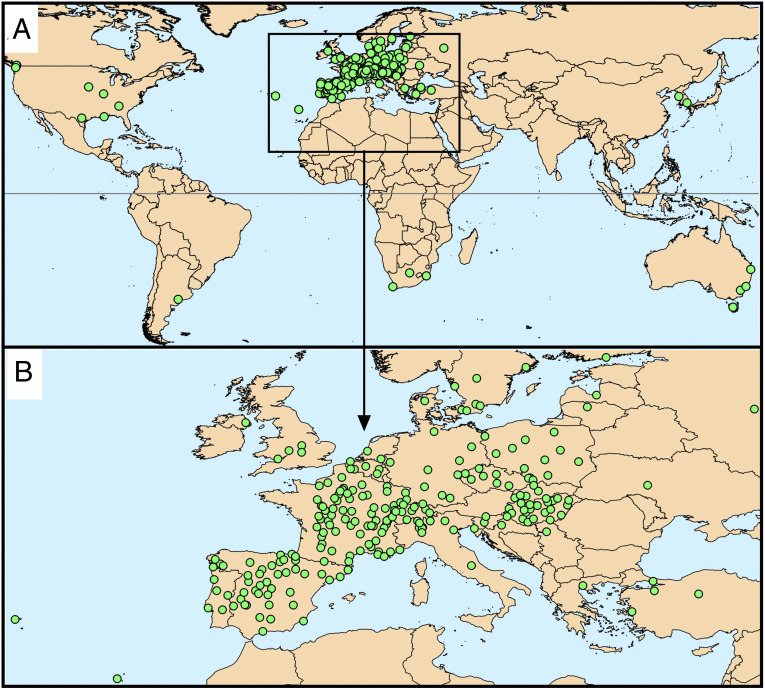
Map of pollen monitoring stations. Green dots represent the 248 monitoring stations from which data were obtained. (*A*) Overview of all stations worldwide. (*B*) Zoom-in on all European stations.

**Fig. 2.**
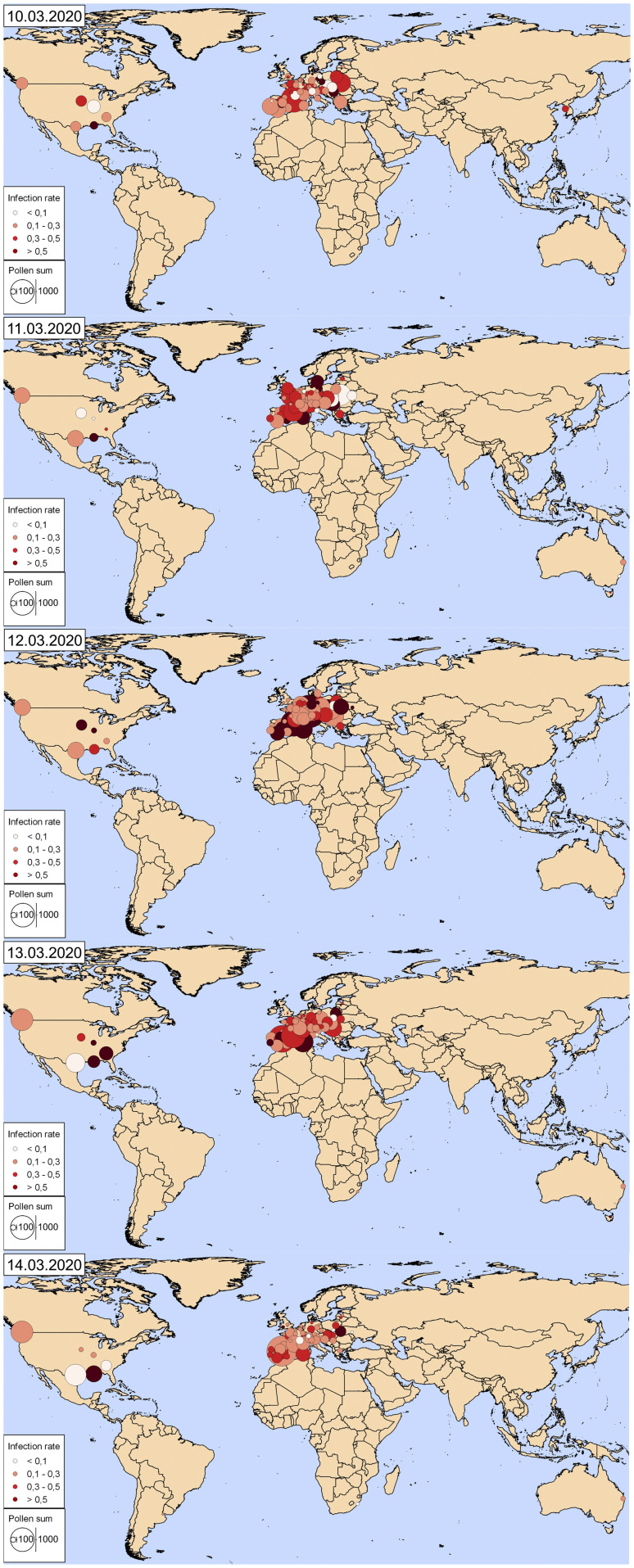
Visualization of pollen concentrations and infection rates during a warm spell during 10 to 14 March 2020. SARS-CoV-2 infection exponential phase (higher infection rates denoted with darker circle color) coincided with higher airborne pollen concentrations (larger circle diameter).

To examine the potential effects of pollen−virus coexposure, a large cross-sectional and longitudinal study was set up, based on 248 airborne pollen monitoring sites, from 31 countries in all inhabited continents across the globe (Fig. 1). The initiative started when, during 10 to 14 March 2020, a warm weather episode brought about higher airborne pollen concentrations across the Northern Hemisphere (denoted as larger circles in Fig. 2), which was evident in mainland Europe mainly on 12 March. This coincided with high SARS-CoV-2 infection rates (denoted with darker color circles in Fig. 2) characteristic for the early exponential infection phase.

**Fig. 3.**
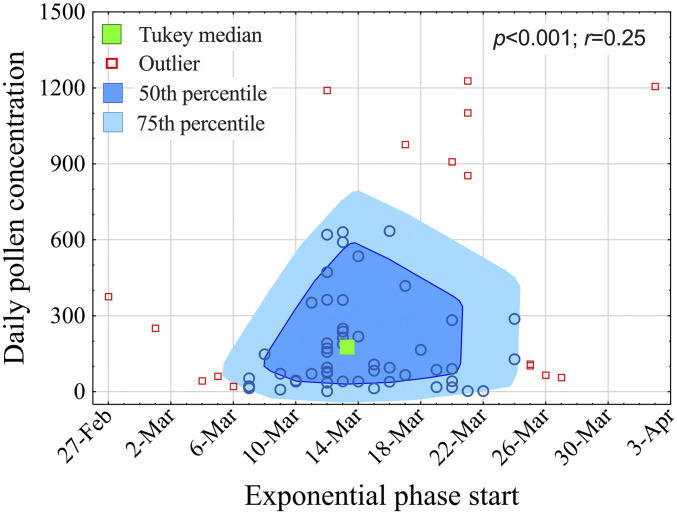
Bag plot depicting the date of onset of SARS-CoV-2 exponential infection phase. Date of onset of the exponential infection phase (*x* axis) across all sites versus the average pollen concentration of the previous 4 d (*y* axis).

The median day of onset of COVID-19 exponential phase (for definition, see *Materials and Methods*) was 13 March 2020 (Fig. 3), which corresponds, on average, to a cumulative pollen concentration of 1,201 grains/m^3^ up to 4 d before (daily average: 240 pollen/m^3^). On a cross-sectional design for all 80 regions under study, it was found that the onset date of the exponential phase per region positively and significantly correlated with the cumulative amount of pollen up to 4 d before (*P* < 0.001, *r* = 0.25). Those regions mainly with lower pollen concentrations and high human contact because of the carnival events in late February, as well as with humid, colder continental climates (on 20–21 March), were categorized as outliers in Fig. 3.

**Fig. 4.**
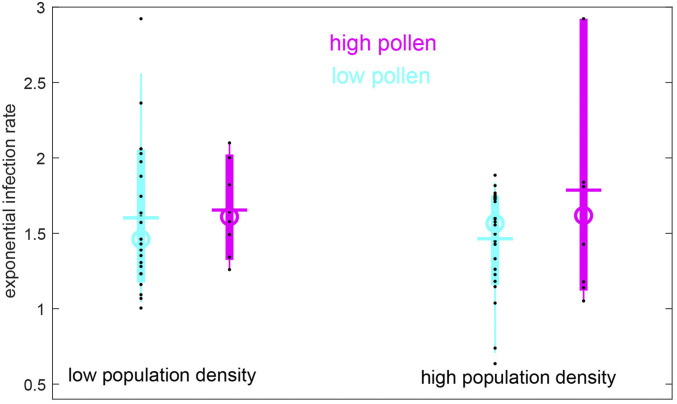
SARS-CoV-2 infection rates are positively correlated with airborne pollen. Mean infection rate in the exponential phase for sites with low (<1,000 inhabitants/km^2^) and high (≥1,000 inhabitants/km^2^) population density and for low (<250 pollen/m^3^) and high (>250 pollen/m^3^) average pollen concentration during the 2 wk of near-constant infection rate. Only the regions and time intervals with no lockdown were selected.

On a cross-sectional approach, we investigated for differences during the exponential infection phase between the infection rates for all sites of the study, grouped into four categories: low vs. high population density and low vs. high pollen concentrations (Fig. 4). To isolate the genuine pollen effect, we elaborated only intervals for all countries without any lockdown. The mean and median of the infection rates were found to differ between low- and high-pollen sites by ∼0.1 (low population density) and 0.3 (high population density); that is, a more pronounced pollen effect was observed for the high-population density sites. The extreme values revealed an even stronger signal: Regardless of the population density, near-zero infection rates were observed only in regions with low pollen levels. Conversely, the absolute maximum infection rate was reached in the high-population vs. high-pollen case (*P* < 0.01).

**Fig. 5.**
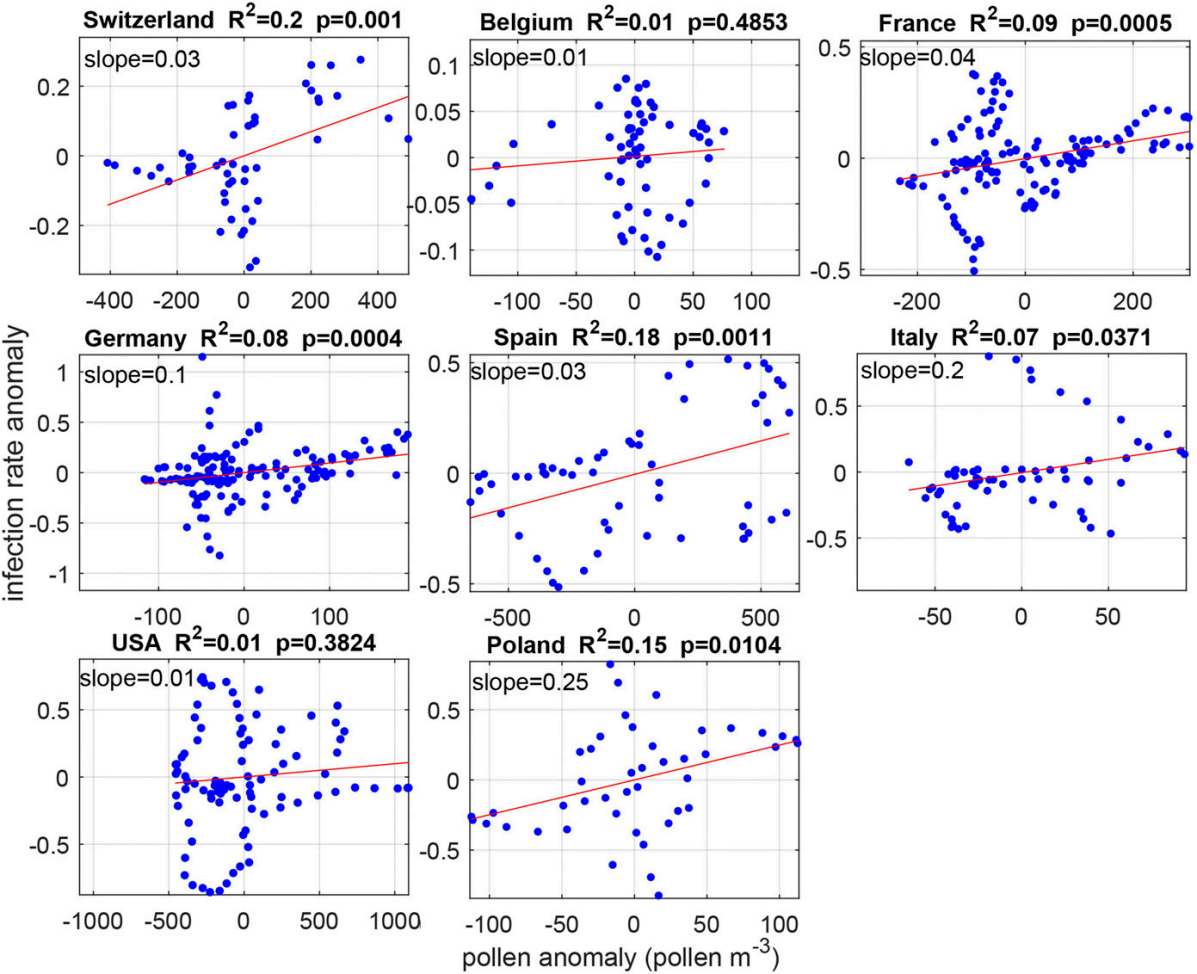
SARS-CoV-2 infection rates are positively correlated with pollen concentrations in a longitudinal data analysis. Per site, anomalies of infection rates are plotted against anomalies in pollen concentrations (blue dots). The slope of the regression line represents the magnitude of the sensitivity of infection rates to pollen concentrations (infection rate per 100 pollen/m^3^). Note the different scales in the panels, both in *x* axes and *y* axes. Only the regions from the geographically large or the bioclimatically diverse countries are analyzed here.

On a longitudinal setup and focusing on the geographically large or climatically diverse countries, which contain the vast majority of regions under study, we investigated for spatial anomalies of the infection rates, which were correlated country-wise with spatial anomalies of pollen concentrations. To eliminate low-level statistical noise, very low pollen concentrations (<50 pollen/m^3^) and regions sparsely populated (<100 inhabitants/m^2^) were not included in the analysis. Only the before or no lockdown time intervals were included in the analyses. It was found that the anomaly correlation coefficient was positive for all countries and significantly positive in six out of eight (Fig. 5). The regression slopes show that the infection rate’s sensitivity to pollen, on average, is 0.04 per 100 pollen/m^3^ (range: 0.03 to 0.25) for the countries with significant correlations. Depending on the region (note the different *x* axes values in Fig. 5), this corresponds to 6 to 15% of the exceedance of the rate over zero. The *R*^2^ values shown in Fig. 5 (including also nonsignificant relationships) illustrate that 10% of variability in the infection rate is explained by its sensitivity to pollen fluctuations.

The pollen effect was proven strong, sometimes regardless of the population density. Switzerland, as one of the countries with the highest pollen concentrations across the world during the exponential phase of the pandemic, serves as a case study to illustrate the relative importance of the pollen effect, by comparing three cities located close to each other and with comparable climates and population densities, but with different pollen exposure (*SI Appendix*, Fig. S1).

**Fig. 6.**
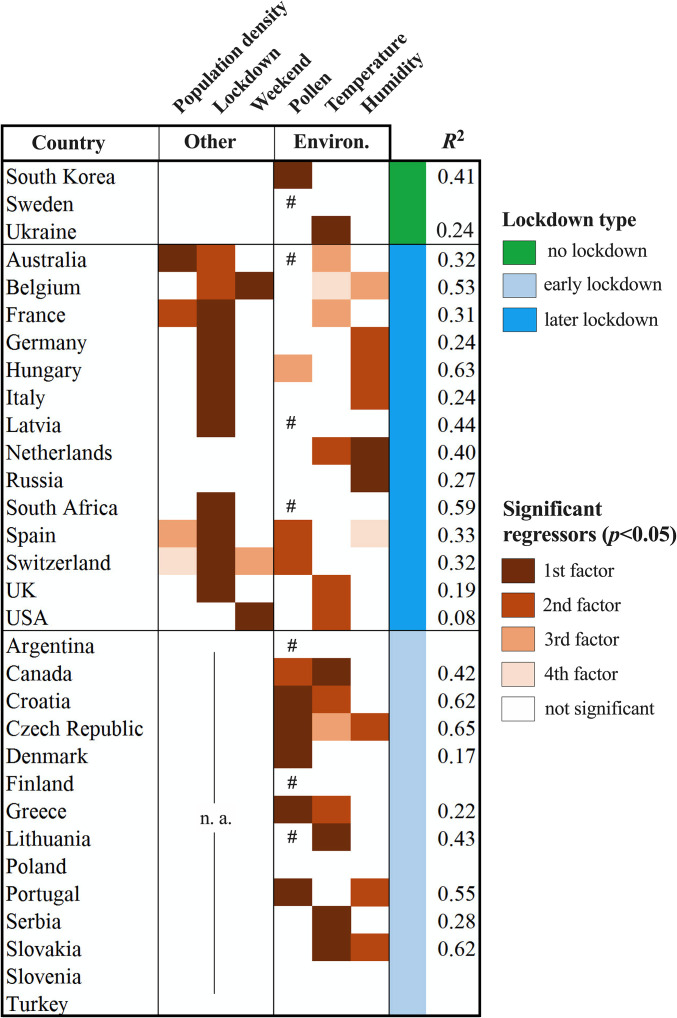
Heatmap of environmental factors and social contact indicators significantly correlating to SARS-CoV-2 infection rates in a longitudinal data analysis. The color reflects the ranking of the variable based on the stepwise removal procedure (ridge regression). Pollen, daily pollen concentration (pollen per cubic meter); Temperature, diurnal temperature range (DTR); Humidity, diurnal humidity range (DHR); Three lockdown regimes are examined: no lockdown at all (green color); mixed, firstly with no lockdown and under lockdown later (blue color); and almost exclusively under lockdown (light blue color). All relationships of infection rates were, by rule, positive with pollen, temperature, and relative humidity, and negative with the weekend and lockdown effects); n.a.,: lockdown not included as variable in the ridge regression; #, total pollen during the study period per region < 5% of the averaged total pollen of all examined regions.

To test the influence of other cofactors, environmental but also human interaction related, we performed a per-country longitudinal analysis (Fig. 6). Complementing the analysis and results in Fig. 5, ridge regressions were conducted for all 31 countries and 130 regions under investigation. For those countries in which no lockdown had been implemented, or the lockdown had started almost in parallel with the onset of the exponential infection phase (<5 d difference), we could not possibly consider the lockdown variable in the analysis. Despite the significant and negative effect of lockdown in the majority of countries for which we included it as dummy variable (11 out of 14 countries, in the mixed design with no lockdown−lockdown regime), environmental cofactors were still significantly correlated with increases in daily infection rates in 12/14 of cases (*P* < 0.05) (Fig. 6). Regardless of the exposure conditions, either with or without a lockdown regime (Fig. 6), of the three environmental factors examined here, pollen was significant in 10/21 countries, air temperature in 14/23, and relative humidity in 10/23. All significant correlations of infection rates with environmental factors (pollen, temperature, humidity) were, by rule, positive, and those with lockdown and weekend, by rule, negative. The average lag effect of airborne pollen on daily infection rates was 4 d (using backward stepwise removal of independent variables), which is consistent with the cross-sectional analyses described above. Under an early lockdown design (lockdown before or <5 d after the onset of the exponential infection phase), pollen concentrations were still significantly and positively correlated with daily infection rates in 6/14 countries, and, in 5/14 pollen, was the primary factor. Under a mixed lockdown design (full exposure ≥ 5 d, then lockdown), lockdown was significantly and negatively correlated with daily infection rates in 11/14 of cases, in 9/14 as the primary factor. Strikingly, even under an early lockdown, the synergy of environmental factors could explain, on average, 44% of the infection rate variability in 9 out of 14 countries (Fig. 6). It is worth mentioning that, of the remaining countries with no significant relationships with airborne pollen abundances (or with other environmental factors as well), 7 countries exhibited very low pollen concentrations during the examined period, explicitly less than 5% of the averaged total pollen load of all countries. These countries, by rule in the Southern Hemisphere or in colder and humid continental climates (Fig. 6), most frequently did not correlate with any environmental parameter at all.

**Fig. 7.**
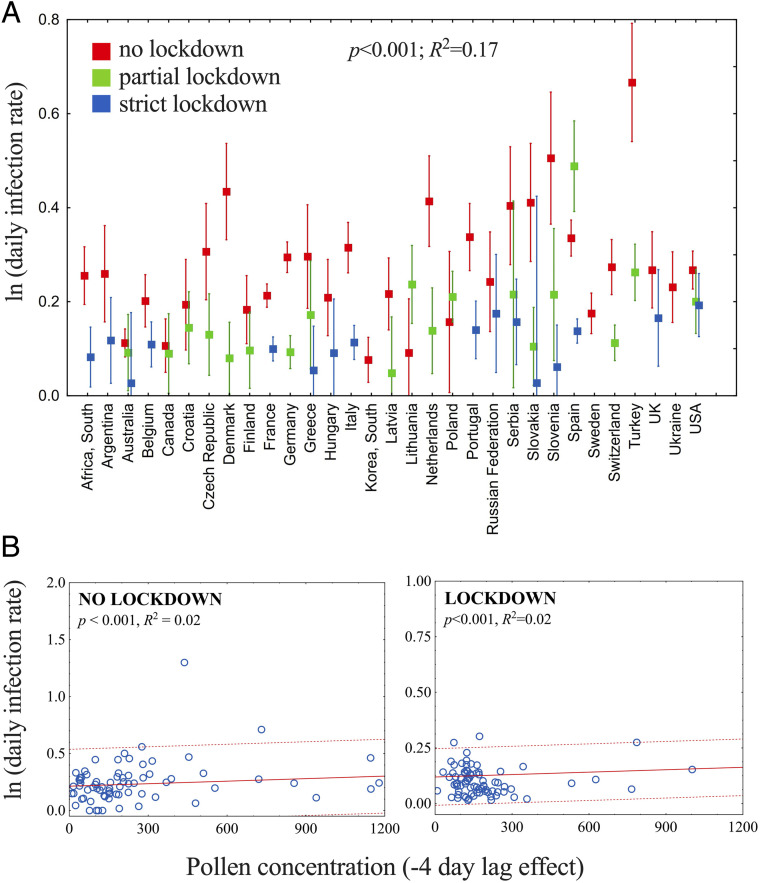
Effect of lockdown on the relationship between pollen concentrations and SARS-CoV-2 infection rates. (*A*) Factorial ANOVA of daily infection rates between lockdown effect and different countries. (*B*) Linear regression of daily infection rates (note the different *y* axes, double in the no-lockdown regime [*Left*]) against pollen concentrations, grouped by lockdown. Only the exponential infection phase and only sites with the lockdown having started not too early (>5 d after the onset of the exponential phase) were included (18 regions, i.e., 26% of the Northern Hemisphere sites).

We further investigated the lockdown effect, longitudinally, among countries, and, cross-sectionally, in association with airborne pollen concentrations. Almost all countries had a lockdown of some type, mostly a partial one. Only nine countries adopted a strict lockdown from the beginning. Lockdown significantly decreased the infection rates as compared to no lockdown (*P* < 0.001) (Fig. 7*A*). A significant positive correlation between daily infection rates and daily pollen concentrations was observed under both lockdown and no-lockdown regimes (*R*^2^ = 0.02; *P* < 0.001). However, the magnitude of the lockdown effect was such that, under comparable amounts of pollen, daily infection rates were reduced to approximately half during lockdown compared to full exposure: The association of infection rates with pollen concentrations was still positive and significant (note the different *y* axes in Fig. 7*B*).

## Discussion

Our large-scale retrospective data analysis based on 80 individual time series from 130 regions in 31 countries in all inhabited continents across the globe (8,019 data points) enabled us to reveal a robust and significant positive correlation between SARS-CoV-2 infection rates and airborne pollen concentrations, which was halved under lockdown. We managed to obtain pollen data from the majority of all pollen monitoring stations worldwide that were operative despite considerable spread of COVID-19 infection rates already by that time, resulting in the most comprehensive aerobiological dataset possible to conduct such a study.

In the current pandemic situation, SARS-CoV-2 infection spread is primarily and foremost dependent on person-to-person interaction, which is mirrored by the observed, significant effect of lockdown. The rapid kinetic of infection in the absence of herd immunity is prone to mask any potential effect of environmental cofactors that may exacerbate contact-dependent mechanisms. The example of Switzerland shown in *SI Appendix*, Fig. S1 highlights the major assumption made in the longitudinal study: The cities should have similar weather conditions and be similar from a sociodemographic standpoint. On the opposite side of this case study, in the United States, these very requirements were not upheld for the five sites tested (distance between them exceeded 2,000 km, some were in maritime and some in strongly continental climate, different states with different strategies regarding lockdown, mean income, and other factors). This lack of homogenous conditions may easily explain the strong scatter in the United States anomaly correlation chart.

The COVID-19 pandemic hit Europe and North America during springtime, when rising air temperatures are associated with increased social and outdoor activities, which, in turn, means increased environmental exposure—to bioaerosols, pollutants, or infected humans. Given the complexity of intertwined environmental, social, and political cofactors, it is anticipated that no clear signal may be observed unless it is tremendously robust. Moreover, environmental exposures, whether climatic factors, air pollutants, or pollen, often exert their effects at the same time, and many of these factors are collinear, which complicates the statistical analysis. Nonetheless, from all the countries that showed a significant correlation of the infection rate with pollen, this correlation was always positive, which suggests that the mechanism reported for pollen exposure on antiviral immunity to rhinovirus (15) could also be influencing innate immunity toward SARS-CoV-2. To verify this statement, we conducted multiple tests to check for bias, including bootstrapping and permutation tests. If, under this statistical noise, we can still see such a signal, we may safely consider the results robust enough, with our concerns being actually about whether we potentially underestimate the magnitude of this effect.

Infections with endemic coronaviruses (strains OC43, HKU1, 229E, and NL63), as well as other frequent respiratory viruses, such as respiratory syncytial virus and influenza A, peak in winter or early spring; a general negative trend of air temperature on these infections has been evidenced (18). Therefore, it is likely that parameters like air temperature act, in the long term, as confounding factors for the short-term positive effect of pollen on infection rates. Also, while the anomaly correlation between airborne pollen and infection rates was significantly positive, the effect size was small, indicating that pollen is only one of a number of environmental factors influencing SARS-CoV-2 infection. However, if one considers that the study was conducted marginally in the start of the pollen season in most regions, this statement may be under dispute. Extending this study deeper into the 2020 pollen season would not offer clearer information, as we would have an even wider variety of data, with ceased lockdown measures and opening borders and tourist activities taking place almost up to the end of 2020.

When checking for additional environmental cofactors, including human interaction indicators, an average of 4 d of lag effect was found in increases in pollen concentrations associated with increases in infection rates. This was connected with the temperature and/or humidity lag of the same or the previous day. A 4-d lag effect of pollen is in agreement with the proposed physiological mechanism of action, an interference of pollen with the innate antiviral immune system. A study based on infection data from Singapore and the Chinese provinces of Tianjin and Hubei estimated an incubation time for COVID-19 of between 4 and 5 d (19, 20), which is much shorter than original estimates (2) but close to our results. It is also in agreement with a hypothesis of environmental exposure factors acting by reducing the incubation period. Unfortunately, this assumption could not be supported by similar pollen data from China, as aerobiological monitoring there is not yet well established.

Respiratory and olfactory epithelium has been shown to express the viral entry receptors for SARS-CoV-2, ACE-2, and TMPRSS2 (21, 22), which makes the nasal cavity a potential early virus reservoir and stresses its importance in innate antiviral defense (23, 24). Since the upper airways are also the entry site for pollen grains, the previously shown immunosuppressive effect of pollen on respiratory epithelia (15) could influence the susceptibility to SARS-CoV-2 infection as well. Pollen grains act on the very site of virus entry, the nasal epithelium, by inhibiting antiviral λ-IFN responses (15). Early treatment with IFN-λ has recently been discussed as a first-line therapeutic option to prevent COVID-19−associated cytokine storm (25–27). This highlights the conclusiveness of our primary hypothesis, which is supported by the epidemiological results reported here.

The observed correlation of airborne pollen with infections did not depend on the allergenic nature of the pollen types present in the air during the study period. Although we analyzed the entire biodiversity spectrum of pollen taxa (*SI Appendix*, Fig. S2), when stratifying pollen by “allergenic” and “total” pollen, both showed similar correlations with COVID-19 cases (*SI Appendix*, Fig. S3). This agrees with our previous findings on immune modulatory effects of pollen, for example, inhibition of NF-κB (28), MyD88 (29), and antiviral IFNs (15), which do not depend on pollen-derived allergens and are effective in sensitized as well as in nonsensitized individuals (30, 31). Thus, although we do not (and could not possibly, to our knowledge) have any information on the allergy status of the COVID-19 cases on which our analysis was based, we assume that the pollen effect is relevant for the entire population. It might, however, be more pronounced in allergics, asthmatics, or chronic rhinosinusitis patients, due to an intrinsically weaker antiviral immune response (32–35).

Our results were not yet able to reveal the genuine magnitude of the pollen effect, as the entire springtime pollen peak of the Northern Hemisphere was not fully included, either in terms of abundance or in its whole seasonality. The data acquisition was stopped in early April due to lockdown restraints. An unavoidable major limitation of the longitudinal data analysis is, therefore, the shortness of some of the time series. During that time, only a few studied sites were subjected to the substantially varying pollen load similar to that shown for Switzerland; practically, we had to deal with two subsets of data, one with a mixed design of lockdown−exposure effects and another design of early enough lockdown to almost annihilate the pollen effect in some occasions.

The sites located in the Southern Hemisphere were mostly out of the pollen season during the study period, and most had not reached the exponential infection phase yet. Whether this is in support of our hypothesis cannot be conclusively answered at this stage, but it should become evident by examining the Southern Hemisphere's pollen season in October 2020 and thereafter.

Another limitation is the spatial resolution of the COVID-19 cases, as, for some sites, local COVID-19 data (*SI Appendix*, Table S1) were not yet available, data had gaps or were registered in a biased way, or the number of cases was too low. In such occasions, we had to access the COVID-19 cases per country, which might not be the best approximation and is reliant on testing strategies within each country. At this early stage of the pandemic, infection rates were based on documentation of numbers of cases presenting to public hospital services and may not have included mild or asymptomatic cases in the community.

To minimize bias of COVID-19 data due to registry lags and errors, we regularly updated our database (last update: 10 May 2020). In most countries, COVID-19 databases were updated within the time frame of a month and then did not change any more. Therefore, we consider our COVID-19 database curated up to 8 April as “reliable.” We were, however, unable, at this stage, to correct for every possible confounder, such as underreporting or changes in testing strategy. In our cross-sectional analysis, we controlled for population density, but we are aware that, still, a comparison across all countries is problematic due to the above limitations, and we attempted to overcome this by doing longitudinal analyses per country, and by two different approaches.

We specifically searched the data, per site and per country, for weekly cycles that might arise from gaps in weekend recordings. While recurrent accumulations of COVID-19 cases on some weekdays, mainly on Wednesdays and Thursdays, can be most likely attributed to weather events, we still included “weekend” as a dummy variable in the ridge regression, where it turned out to be less significant than the effects of lockdown and environmental factors, with the exception of three countries.

In the light of the present pandemic situation, our findings should be communicated with caution so as to avoid misunderstandings and panic. It has to be made very clear that 1) the demonstrated correlations suggest that pollen is a modulating factor to the overall progression of the SARS-CoV-2 infection, with the potential to add an extra 10 to 30% to the infection rate (Fig. 5), 2) there is no evidence for airborne pollen grains themselves being carriers of virus particles (36), and 3) without contact, there is no risk of infection.

Of note is that the effect of pollen on reported infection rates was shown to be less pronounced under lockdown regimes. It is also possible that high temperatures in summer would counteract infections to some extent, provided, of course, that social distancing will still be kept. Therefore, the infection-promoting effect of pollen could become evident only during spring, when air temperatures are not high enough yet to limit viral spread, but high concentrations of tree pollen occur. To avoid future waves of high virus transmission under “favorable” combinations of air temperature, humidity, and pollen, we recommend taking stricter protection measures, for example, wearing particle filtering masks during springtime higher pollen concentrations. The installation of reliable, real-time bioaerosol measurement networks and the use of pollen information and forecasting systems should be encouraged.

Looking to the future, it is yet unknown whether other air particles, like fungal spores, or complex interactions with pollen, other meteorological variables, and air pollutants may also play a role. Even though there is published evidence on the effects of various environmental parameters, like nitrogen dioxide (NO_2_), particulate matter (PM_2.5_), and ultraviolet radiation (37–41), these usually refer to preliminary results and investigation of only a single factor. If one takes into account the huge effect of ongoing climate change and urbanization on the long-term trends in airborne pollen levels (42, 43), as well as emerging viral infections, it is of utmost importance to forecast the associated risk for human health in future pandemics and take appropriate measures to reduce it as much as possible. Coexposure is certainly not the exception but the rule under natural conditions, and, hence, we strongly suggest that modeling and forecasting of ongoing and future pandemics ought to consider the whole “soup” of exposome.

## Materials and Methods

Following the strictest publishing recommendations during the COVID-19 pandemic, we followed the STROBE (Strengthening the Reporting of Observational Studies in Epidemiology) protocol, as follows.

### Experimental Design.

To test our primary hypothesis that coexposure to airborne pollen enhances the susceptibility to infection with SARS-CoV-2, we performed a large-scale retrospective, cross-sectional and longitudinal data analysis on daily SARS-CoV-2 infection rates and the environmental cofactors of airborne pollen concentrations, air temperature, and relative humidity. Apart from environmental cofactors, estimates of human-to-human interaction were also considered, that is, population density, lockdown dates, and lockdown strictness. The entire study period was from 1 January to 8 April 2020. Longitudinal data analysis was restricted to the exponential infection phase as determined per site, ranging, on average, from start/middle of March to 8 April 2020 (median = 22 d).

### Data Sources.

Airborne pollen concentrations were obtained via monitoring stations across the globe. So as to have representative sites from different climatic regions, we collected data from a total of 248 aerobiological monitoring stations across the world (Fig. 1), mostly operating Hirst-type volumetric traps, following the standard operating guidelines (44).

The climatic classification of participating countries was performed using the Köppen−Geiger classification (45). Daily COVID-19 cases were retrieved for a total of 80 regions (compiled from 130 sites) in 31 countries and on five continents as reported by the local governmental authorities. Whenever available, we chose the finest possible spatial resolution of COVID-19 data, that is, on the level of state, county, or metropolitan region, to best match the pollen data (*SI Appendix*, Table S1). Last data access was on 10 May 2020.

Air temperature and relative humidity values were obtained from the open-access European Centre for Medium-Range Weather Forecasts Reanalysis 5 meteorological reanalysis. Data were processed per grid point, with the regional average being extracted by point (pixel) or polygon (shapefile). Data on population density was retrieved from the Demographic Yearbook of the United Nations Statistics Division (UNSD) (https://unstats.un.org/unsd/demographic-social/sconcerns/popsize/). For some metropolitan regions that were not listed by the UNSD, we searched Wikipedia.org. Dates of major national and regional lockdown measures were retrieved by extensive internet searches, starting from Wikipedia.org and following the sources cited therein, such as official announcements made by the local governments.

### Data Preprocessing.

Of the data initially acquired from the 248 pollen monitoring stations across the globe, we further analyzed data from 130 regions, from different climatic regions, from humid subtropical to arid Mediterranean, temperate, oceanic, and continental climates (*SI Appendix*, Table S1). The selection of the sites was based on data availability of COVID-19 cases by that time. From the pollen concentrations (pollen grains per cubic meter of air) per plant taxon and station, we calculated daily pollen total concentrations by summing up all pollen on that specific day, but excluding fungal spores. So as to ensure harmonized data across all monitoring stations, when acquiring the pollen data, we clearly and necessarily instructed pollen data providers to provide their data only if they genuinely classify the whole spectrum of the biodiversity in each site. In locations that this was not the case, we did not consider them in the final analysis. So, practically, what we analyzed in this design is truly the whole spectrum of pollen taxa, which, in many sites, accounted for a total number of more than 20 taxa already by 8 April 2020 (depicted in *SI Appendix*, Fig. S2).

Regarding COVID-19 cases, so as to harmonize the registered cases (especially for the cross-sectional analysis), we used daily exponential infection rates (46), calculated from daily COVID-19 cases as follows:

$$\left(DIR\right)=\ln\left(\frac{\sum_{k}^{n}}{\sum_{k}^{n-1}}\right),$$ [1]

where *DIR* is the change in infection rate on day *n*, $\sum_{k}^{n}$ is the sum of confirmed COVID-19 cases from the calendar day *k* of the first case until calendar day *n*, $\sum_{k}^{n-1}$ is the sum of confirmed COVID-19 cases from the calendar day *k* of the first case until the calendar day *n*-1, and *k* is the calendar day of the first coronavirus case per region.

From extracted raw data of temperature and relative humidity, we calculated diurnal temperature ranges (DTR = T_max_ – T_min_) and diurnal humidity ranges (DHR = RH_max_ – RH_min_). By use of DTR and DHR, we attempted to take into account circadian patterns in pollen production and dispersion and, simultaneously, reduce the number of independent variables and lower multicollinearity.

The date of onset of exponential infection phase was defined per site as per all the following criteria: 1) to avoid nonlocal transmission, adequate number of confirmed coronavirus total cases by this date with a minimum of 100; 2) to avoid registration errors, beyond the above threshold, higher than linear increase for at least three successive days; 3) to avoid artificial “jumps” because of improved registration efficiency and so as to avoid the artificial first high peak of infection rates (as per the kinetics of Eq. **1**), selection of the second of the above three successive days with higher than linear increase.

### Cross-Sectional Data Analysis.

Combining data from all sites, we used general linear models (GLMs) and one-way and factorial ANOVAs and simple linear regressions, to test for overall correlations between SARS-CoV-2 infection rates and airborne pollen, controlling for 1) population density or 2) lockdown effect. To select for appropriate lag effects, especially of pollen, we ran autoregressive models and assessed the cross-correlations of the above-mentioned variables, as in ref. 15. To additionally check for the linearity of the relationships, generalized nonlinear models were also tested, in exactly the same context. The selection of the GLMs in the final analysis was based on the residual analysis per region, which determined whether each regional dataset followed the normal distribution assumption. For the visualization of results, box−whisker plots were used for the extremes of pollen concentrations vs. infection rates, grouped by high vs. low population density. Moreover, we applied bag plots using the Tukey median depth (47) (onset date of the exponential phase of the pandemic per region correlated with pollen concentrations of the previous 4 d), paralleled with one-way ANOVA and Pearson correlation to validate the relationship. Last, we used scatterplots with linear regression fits with the respective CIs to express significant slopes and quantify the pollen and lockdown effects.

### Longitudinal Analysis.

Per country, we included only the time period for which the infection phase and airborne pollen peak overlapped (median: 22 d). The analyses were restricted to the level of country, as lower resolution included microclimatic variability, which increased the statistical noise and made any signal very weak to detect. However, for each country, all possible data points were included from different regions, when more than one of the sites were involved. GLMs and autoregressive models with multiple independent variables were run per country to test for multiple effects on infection rates. In all cases, a backward stepwise removal of independent variables was applied. Significance levels at the 95% interval, as well as the coefficient of determination and residual analysis, determined the robustness of the results obtained. Furthermore, for geographically larger countries, anomaly correlations were employed, as in similar analytical designs with high spatial statistical noise (48), to make a sensitivity analysis of pollen alone on its impact on infection rates. Finally, ridge regressions were conducted per country, using a backward stepwise removal of independent variables, to test for relationships of infection rates with 1) lockdown, 2) weekend registration underestimation errors, and 3) environmental factors (DTR, DHR, and pollen concentrations), including lag effects of 0 to −5 for all continuous variables. Ridge regression are well known for dealing with multicollinearity issues, and partial correlations aid in identifying the most significant parameters and their lag effects and synergistic effects among independent variables, as well as confounding factors (49). The dummy variable of “lockdown” was only included for all countries that had at least 5 d of “no lockdown” prior to the lockdown during the exponential phase, so as to have enough data points for the analysis. As, on several occasions, the reporting of COVID-19 daily cases was biased toward lower numbers during the weekends, we inserted a dummy variable for the weekend effect, to control for such artificially reduced registries in some countries. For the visualization, a heatmap was generated to identify the associated effects of various cofactors on infection rates. Maps were created per occasion using QGIS 2.4.0 (https://qgis.org/en/site).

All analyses were performed by use of either the software Statistica 13.3 (TIBCO Software Inc.) or R scripts (see below).

### Methods against Bias.

To minimize bias across all levels of the study, we elaborated on the following.

#### Selection bias.

##### Airborne pollen data.

The sites originally selected practically corresponded to all active pollen monitoring stations in the world, as many do not operate at all in winter months. Also, data acquired initially were screened for large data gaps (more than three successive days within the exponential phase of the pandemic spread) and for including the whole spectrum of pollen taxa expected in an average site (harmonization of pollen measurements). The spanning period had to be from as early as possible in 2020 (most frequently, on 1 January 2020) and mostly up to 8 April 2020. Pollen data beyond that date were not acquired, as 1) Hirst-type (or Rotorod-type) measurements are based on manual and laborious methods, and, hence, data are delivered often with a delay of at least 8 d; and 2) because of the lockdown restrictions across the world, often strict, many of the monitoring stations suspended their operation. Obtaining data only from a few would eliminate the harmonization of data and minimize the globality of the study design as well as the possibility to investigate climatic variability. Those sites that did not satisfy any of the above prerequisites were excluded completely. The first screening accounted for a sum of 248 sites. In each one of these sites, we summed up all different pollen types each day to obtain the daily pollen load that could affect the spread of the viral infections. To avoid microclimatic spatial variability and potentially obtain clear signals, when many pollen monitoring sites existed per country, we averaged the data over the region (state, province, canton, or county). This depended also on the data availability of daily COVID-19 cases at this scale. The overview of analyzed datasets is shown in *SI Appendix*, Table S1.

##### COVID-19 cases.

Given the spatial and temporal availability of the airborne pollen data and taking into account the availability of COVID-19 infection data, we obtained daily COVID-19 cases per city or metropolitan region, whenever possible, especially considering the frequent clustering of COVID-19 cases in large urban areas. When the per-region choice was not feasible, we switched to per country COVID-19 data, for example, for smaller countries or when regional COVID-19 numbers were too low in comparison to nationwide numbers. Vice versa, in very large countries, we necessarily broke down the COVID-19 cases into metropolitan regions, provinces, or states, so as to reduce the variability in microclimatic and sociological factors. This was the case in the countries of France, Germany, Italy, Spain, Switzerland, and the United States, along with Australia and South Africa in the Southern Hemisphere. The detailed data availability and level of processing are given in *SI Appendix*, Table S1.

#### Confirmation bias.

The data analysis was independently performed by three different groups of data analysts, from different countries, all with different approaches, who compared their findings at regular intervals.

#### Outliers.

To ensure that no “redness” of statistical noise exists (50), which, if categorized as outliers, may reduce the signal (underfitting), box plots were used to identify outliers and extreme values, particularly bag plots, to additionally interpret medians and averages of observations, their distribution, and symmetry (47). Remaining outliers were attempted to be interpreted with additional cofactors, when appropriate. Moreover, intentional outliers were created in the form of dummy variables for contact indicators, like population density and lockdown effects, which could bias the results. Because of registration errors in COVID-19 cases and lack of harmonization across the regions and countries in the study, an additional dummy variable was created, highlighting the effect of the weekend. Variability within each week was thoroughly checked among the weekdays to confirm whether the obtained variability could be a recording error or a potential signal. As, in most sites of the Northern Hemisphere, we found out that the signal was consistent regardless of the country or region examined (i.e., more cases on Wednesdays and Thursdays) and as this could not be further confirmed with local authorities as per the registration accuracy, we preferred to consider that the largest proportion of this variation would be an environmental signal, and we did not further manipulate this.

#### Overfitting.

As a sensitivity analysis, we ran bootstrapping with 1,500 iterations, using the R package {boot}. Bootstrapping was run with different combinations of datasets: 1) the entire dataset, that is, daily infection rates vs. daily pollen concentrations, DTR, and DHR, each including 0- to 5-d lag effects; and 2) daily infection rates and daily pollen concentrations alone (0- to 5-d lags) without the DTR and DHR data. In addition, to test the significance of obtained correlations, we performed permutation tests on the data for the longitudinal analysis using the R package {lmPerm}. To test potential overfitting of the acquired models, ridge regressions were employed, and the backward stepwise technique and the partial correlations of all factors and the significant lag effects of continuous variables were taken into account. For ensuring the robustness and lack of bias in the results, we checked the lambda (λ) values of the regularization (51), from 0.1 to 10^−6^, and the error values did not change, but only to a magnitude of the third decimal. We selected a value of λ = 0.1 so as to ensure a higher strictness in the analysis.

#### Cofactors and confounding factors.

To test for significant cofactors and confounding factors, we conducted ridge regression with a stepwise backward elimination procedure of the independent variables, and we checked the partial correlations to eliminate multicollinearity and select only the genuinely significant variables, especially in the longitudinal analysis.

## Supplementary Material

Supplementary File

## Data Availability

Daily data of 1) pollen concentrations, 2) SARS-CoV-2 infection rates, 3) air temperature, 4) relative humidity, 5) population density, and 6) lockdown dates have been deposited in Mendeley (DOI: 10.17632/6f8y8d9cgw.1) (52).
