# Supplementary material for: Higher airborne pollen concentrations correlated with increased SARS-CoV-2 infection rates, as evidenced from 31 countries across the globe
Source: Proc Natl Acad Sci U S A. 2021 Mar 8;118(12):e2019034118. doi: 10.1073/pnas.2019034118 (PMC7999946; doi:10.1073/pnas.2019034118)
Supplement: Supplementary File [file pnas.2019034118.sapp.pdf]

PNAS
www.pnas.org

Supplementary Information for

Higher airborne pollen concentrations correlated with increased SARS-CoV-2 infection rates, as evidenced from 31 countries across the globe

Athanasios Damialis[1*+], Stefanie Gilles[1+], Mikhail Sofiev[2], Viktoria Sofieva[2], Franziska Kolek[1], Daniela Bayr[1], Maria P. Plaza[1], Vivien Leier-Wirtz[1], Sigrid Kaschuba[1], Lewis H. Ziska[3], Leonard Bielory[4,5,6], László Makra[7], Maria del Mar Trigo[8], COVID-19/POLLEN study group[#], Claudia Traidl-Hoffmann[1]

* Athanasios Damialis
Email: thanos.damialis@tum.de

[+] : Equally contributed
[#] : The list of co-authors (and their full affiliations and e-mails) is attached to the end of the Appendix.

This PDF file includes:

Figures S1 to S3
Supplementary text for Figures S1 to S3
Table S1
References
COVID-19/POLLEN study group co-author full names, affiliations and e-mails

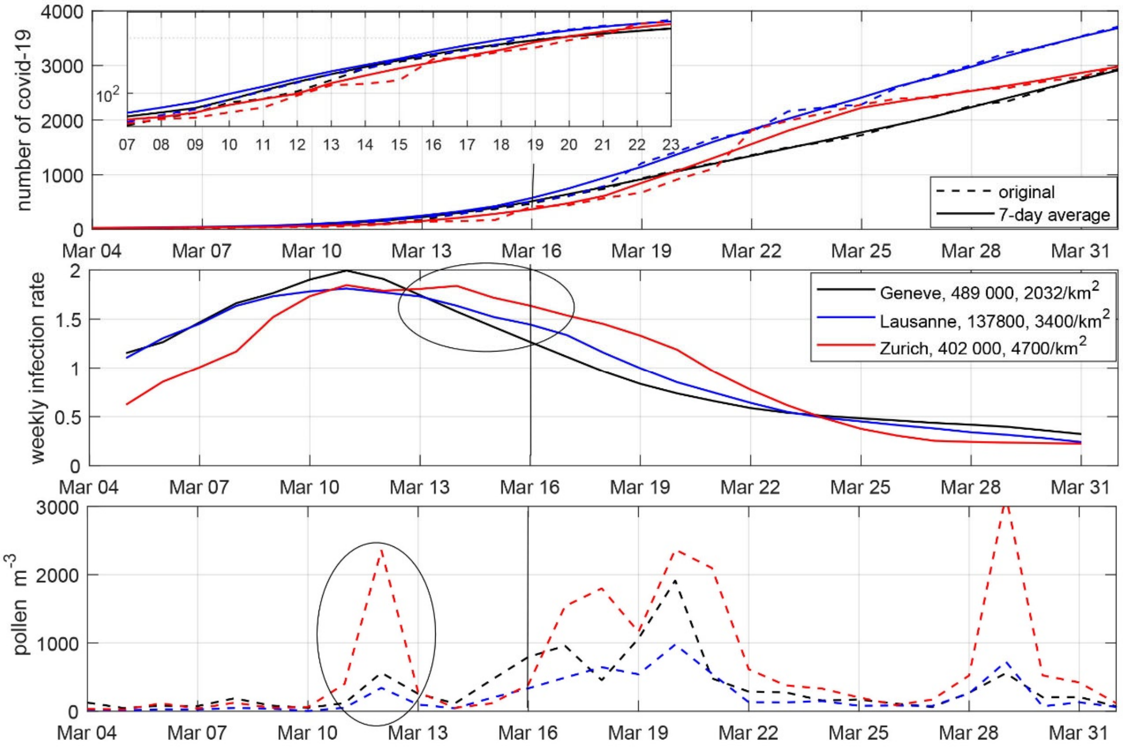

Fig. S1. Switzerland as case study to illustrate the magnitude of the pollen effect. Number of COVID-19 cases, infection rates (7-day moving average) and airborne pollen concentrations (pollen m^{-3}) are shown as a function of date for three Swiss cantons.

Supplementary Information Text for Fig. S1

The example of Switzerland illustrates the relative importance of the effects of pollen, population density and lockdown. Switzerland was one of the countries with the highest pollen concentrations for several days during the exponential phase of the pandemic, which made it possible to compare three cities in Switzerland located close to each other and having comparable climate and population densities, but different pollen concentrations.

The pollen spike on 12 March in Zurich was 5 times that of Geneva and 8 times higher than in Lausanne. As a result, the overall tendency of reduction of the infection rate from 11 March was broken in Zurich (ovals mark the spike and its effect) – and the anomaly faded out only a week later. Of note, the pollen spikes that occurred during the lockdown phase exhibited a by far less pronounced effect.

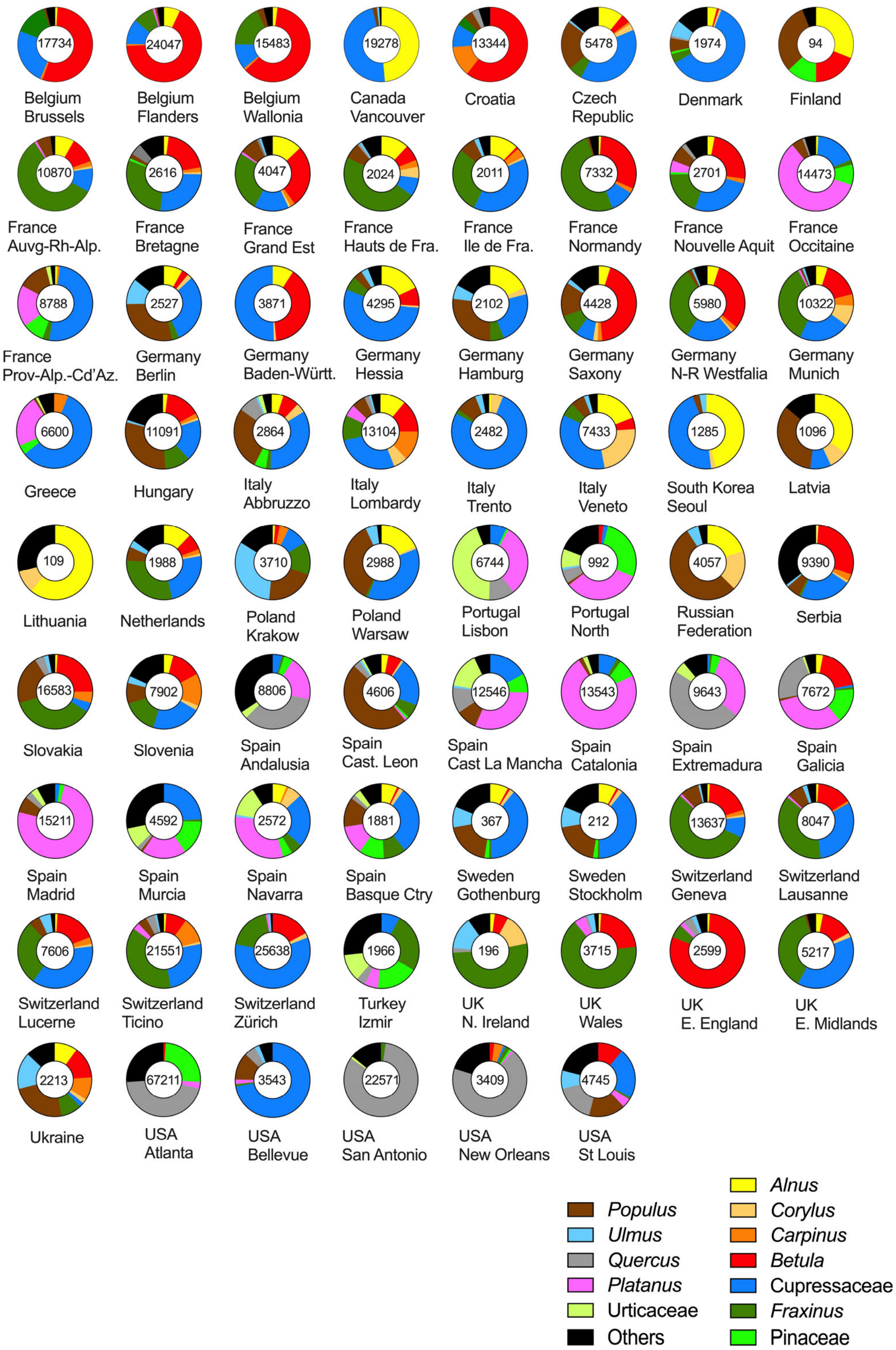

Figure S2. Donut charts of the biodiversity of all monitored airborne pollen per region, in 70 regions across the Northern Hemisphere. Focus was given on allergenic pollen primarily (color depiction), while the rest were denoted as 'Others' (black color depiction). The numbers in the middle of each chart indicate the total number of all pollen grains recorded during the study period for each region.

The Southern Hemisphere has been excluded here, as the daily average pollen concentration was only 17 pollen grains per cubic meter of air, the majority belonging to grass pollen (in contrast to a daily average of more than 200 pollen in the Northern Hemisphere).

Supplementary Information Text for Fig. S2

To assess the biodiversity of the pollen time series we analysed, we show here 70 donut charts (for the Northern Hemisphere), representing the percentages of each pollen taxon as the relative contribution to the overall pollen abundance. We found that the total pollen amount actually refers to mainly allergenic pollen for the study period. The majority of the taxa included in this study refer to allergenic ones, like *Alnus*, *Betula*, *Corylus*, Cupressaceae, and *Fraxinus*. To define which pollen types are ‘allergenic’, we followed published results per pollen taxon, e.g. as in (1) and references therein.

The allergenic taxa are denoted with color, whereas all the rest with black. The black parts are overruled by the color ones and the black pieces in the donut charts may represent a large number of taxa, like *Acer*, Myrtaceae, *Plantago*, *Salix*, but also late spring-summer emerging pollen (not yet present in abundance by the time of the study implementation), like *Ambrosia*, *Artemisia*, Chenopodiaceae, *Olea*, *Plantago*, *Rumex*, Poaceae. Even though the biodiversity is not consistent among regions and countries, with a variable ranking order of pollen taxa, the take-home message is that the non-allergenic or the not-present-yet taxa (‘Others’, denoted as the black parts of the donuts) could be numerous but not abundant at all, yet.

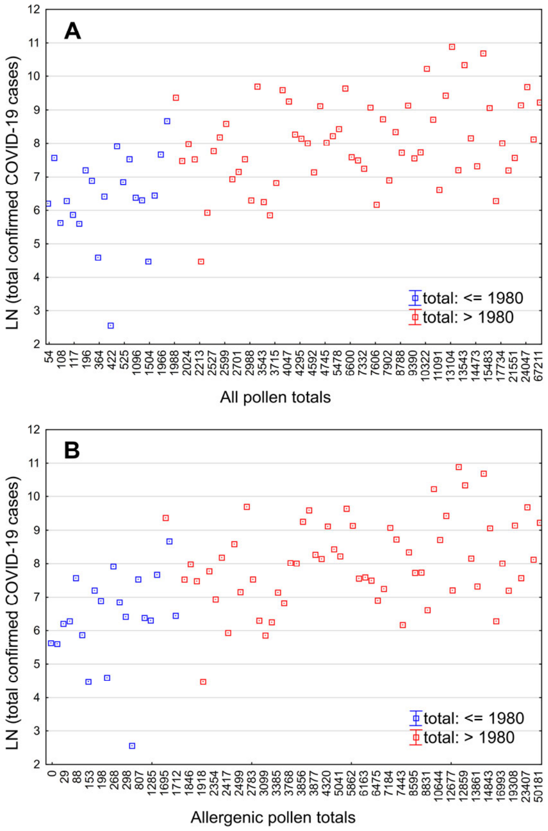

Figure S3. Scatterplot of the distribution of the total confirmed COVID-19 cases in relation to pollen totals. To analyze, in parallel, the infection cases in association with pollen abundances, we differentiated the 1st quartile of pollen data (low concentrations, as in the Southern Hemisphere), which accounted for a threshold 1980 pollen.

Supplementary Information Text for Fig. S3

To evaluate whether the total amount of pollen per region is the most representative and appropriate for our research question, we assessed the whole diversity of airborne pollen taxa for each and every site out of 248 originally acquired and for all the regions and all 31 countries included in the analysis. It is clearly demonstrated that the effect of our originally calculated totals of pollen are almost identical to the newly calculated allergenic pollen by 90.7%. Regarding a potential change in the originally calculated pollen signal in the whole analysis, if we apply a fitting line, the difference of the coefficient of determination is only of the magnitude of 0.03 (significant and positive in both scenarios). The above similarity is simply due to the fact that our study period refers almost exclusively (for the Northern Hemisphere) to airborne pollen from winter-spring trees and shrubs.

Table S1. Overview of data sources for pollen and COVID-19 cases. Data from a total of 130 single aerobiological measurement stations included in the analysis. When more than 2 sites were equally eligible, we picked the site(s) with the highest population. Last data access on 10 May 2020.

Country	Köppen climate	Region of pollen data (*n*)	Region of COVID-19 data	Source of COVID-19 data
Africa, South	Csb	Cape Town (1)	West Cape	https://www.nicd.ac.za/covid-19-update-61/
Africa, South	Cfa	Durban (1)	Kwa Zulu-Natal	
Africa, South	Bsh	Kimberley (1)	North Cape	
Argentina	Cfa	Bahia Blanca (1)	Buenos Aires	https://www.argentina.gob.ar/salud/coronavirus-COVID-19
Australia	Cfa	Brisbane (1)	Queensland	https://www.health.gov.au/news/health-alerts/novel-coronavirus-2019-ncov-health-alert/coronavirus-covid-19-current-situation-and-case-numbers
Australia	Cfb	Australian Capital Territory (1)	Canberra	
Australia	Cfa	Sydney (1)	New South Wales	
Australia	Cfb	Launceston (1)	Tasmania	
Belgium	Cfb	Brussels (1)	Brussels (metropolitan area)	Sciensano: https://epistat.wiv-isp.be/covid/
Belgium	Cfb	Genk (1)	Flanders	
Belgium	Cfb	Tournai (1)	Wallonia	
Canada	Cfb	Vancouver (2)	Greater Vancouver	British Columbia COVID-19 Dashboard: https://experience.arcgis.com/experience/a6f23959a8b14bfa989e3cda29297ded
Croatia	Cfa	Zagreb (1)	Croatia	Ministarstvo zdravstva Republike Hrvatske: https://www.koronavirus.hr
Czech Republic	Dfb	Prague (1)	Czech Republic	https://onemocneni-aktualne.mzcr.cz/covid-19
Denmark	Dfb	Copenhagen (1)	Denmark	Statens Serum Institut: https://www.ssi.dk/aktuelt/sygdomsudbrud/coronavirus/covid-19-i-danmark-epidemiologisk-overvaagningsrapport
Finland	Dfb	Helsinki (1)	Finland	yle.fi: https://yle.fi/uutiset/3-11300232
France	Cfb	Auvergne-Rhone-Alpes (4)	Auvergne/Rhone/Alpes	ARS/Santé Publique France/Ministère des Solidarités/Santé: https://www.ars.sante.fr
France	Cfb	Bretagne (3)	Bretagne	
France	Cfb	Grand Est (3)	Grand Est	
France	Cfb	Hauts de France (2)	Hauts de France	
France	Cfb	Paris (1)	Ile de France	
France	Cfb	Caen (1)	Normandy	
France	Cfb	Nouvelle Aquitaine (2)	Nouvelle Aquitaine	
France	Cfb	Occitaine (2)	Occitaine	
France	Csa	Provence-Alpes-Cote d´Azur (4)	Provence-Alpes-Cote d´Azur	
Germany	Cfb	Berlin (1)	Berlin	Robert Koch Institute: https://www.rki.de/DE/Home/homepage_node.html
Germany	Cfb	Freiburg (1)	Baden-Wuerttemberg	
Germany	Cfb	Fulda (1)	Hesse	
Germany	Cfb	Hamburg-Borstel (1)	Hamburg	
Germany	Cfb	Leipzig (1)	Saxony	

Germany	Cfb	Mönchengladbach (1)	North Rhine-Westphalia	
Germany	Dfb	Bavaria (2)	Bavaria	
Greece	BSk	Thessaloniki (1)	Greece	https://covid19.gov.gr
Hungary	Dfa	Budapest (1)	Hungary	https://koronavirus.gov.hu
Italy	Cfa	L'Aquila (1)	Abruzzo	Ministero della Salute: http://www.salute.gov.it/nuovocoronavirus
Italy	Cfa	Lombardy (5)	Lombardy	
Italy	Cfa	San Michel all' Adige (1)	Autonomous Province of Trento	
Italy	Cfa	Padua (1)	Veneto	
Korea, South	Cwa	Seoul (1)	Seoul	ncov.mohw.go.kr/en
Latvia	Dfb	Riga (1)	Latvia	https://covid19.gov.lv/en/node/16457
Lithuania	Dfb	Siauliai (1)	Lithuania	https://koronastop.lrv.lt/en/
Netherlands	Cfb	Netherlands (2)	Netherlands	Rijksinstituut voor Volksgezondheid en Milieu: https://www.rivm.nl/en/novel-coronavirus-covid-19/current-information
Poland	Dfb	Krakow (1)	Lesser Poland	Serwis Rzeczypospolitej Polskiej: https://www.gov.pl/web/koronawirus/wykaz-zarazen-koronawirusem-sars-cov-2
Poland	Dfb	Warsaw (1)	Warsaw	
Portugal	Csa	Lisbon (1)	Lisbon and Tagus Valley	Direção-Geral da Saúde: https://covid19.min-saude.pt
Portugal	Csb	Porto (1)	North Portugal	
Russian Federation	Dfb	Moscow (1)	Russian Federation	https://coronavirus-monitor.ru/coronavirus-v-moskve/
Serbia	Cfa	Novi Sad (1)	Serbia	Статистика COVID-19 у Србији: https://covid19.data.gov.rs
Slovakia	Dfb	Bratislava (1)	Slovakia	Koronavírus a Slovensko: korona.gov.sk
Slovenia	Dfb	Ljubljana (1)	Slovenia	https://www.gov.si/teme/koronavirus/
Spain	Csa	Andalucia (3)	Andalucia	https://cnecovid.isciii.es/covid19/
Spain	Csa	Castilla y Leon (11)	Castilla y Leon	
Spain	Cfb	Castilla-La Mancha (6)	Castilla-La Mancha	
Spain	Csb	Catalonia (5)	Catalonia	
Spain	Csa	Extremadura (5)	Extremadura	
Spain	Csb	Galicia (3)	Galicia	
Spain	Csa	Madrid (1)	Madrid	
Spain	Bsh	Cartagena (1)	Murcia	
Spain	Bsh	Navarra (3)	Navarra	
Spain	Cfb	Basque country (3)	Basque country	
Sweden	Dfb	Gothenburg (1)	Västra Götaland	https://www.folkhalsomyndigheten.se/smittskydd-beredskap/utbrott/aktuella-utbrott/covid-19/
Sweden	Dfb	Stockholm (1)	Stockholm	
Switzerland	Cfb	Geneva (1)	Geneva	https://www.bag.admin.ch/bag/en/home/krankheiten/ausbrueche-epidemien-pandemien/aktuelle-ausbrueche-epidemien/novel-cov.html
Switzerland	Cfb	Lausanne (1)	Lausanne	
Switzerland	Cfb	Lucerne (1)	Lucerne	
Switzerland	Cfb	Ticino (2)	Ticino	
Switzerland	Dfb	Zürich (1)	Zürich	

Turkey	Csa	Izmir (1)	Izmir	https://covid19.tubitak.gov.tr
Ukraine	Dfb	Vinnitsya (1)	Ukraine	Department of Health and Rehabilitation of Vinnytsia Regional Council, Ukraine
United Kingdom	Cfb	Leicester (1)	East Midlands	https://www.gov.uk/guidance/coronavirus-covid-19-information-for-the-public
United Kingdom	Cfb	Ipswich (1)	East of England	
United Kingdom	Cfb	Belfast (1)	North Ireland	
United Kingdom	Cfb	Cardiff (1)	Wales	
USA	Cfa	Atlanta, Georgia (1)	Georgia	https://www.cdc.gov/coronavirus/2019-ncov/cases-updates/cases-in-us.html
USA	Dfa	Bellevue, Nebraska (1)	Nebraska	
USA	Cfa	Lackland, San Antonio, TX (1)	San Antonio, Texas	
USA	Cfa	New Orleans, Louisiana (1)	New Orleans, Louisiana	
USA	Cfa	St. Louis, Missouri (1)	St. Louis, Missouri	

References for Supplementary Information

1. A. Damialis *et al.*, Climate Change and Pollen Allergies. In: M. Marselle, *et al.* (eds). *Biodiversity and Health in the Face of Climate Change*. Springer, Cham, pp. 47-66 (2019).

COVID-19/POLLEN study group: Co-author names, affiliations and e-mails.

First Name	Last Name	Affiliation	Country	Email	ORDER
Gilles	Oliver	RNSA (French Aerobiology Network), Brussieu, France	France	gilles.oliver@rnsa.fr	14
Nhân	Pham-Thi	RNSA (French Aerobiology Network), Brussieu, France	France	drphamthi@gmail.com	15
Michel	Thibaudon	RNSA (French Aerobiology Network), Brussieu, France	France	michel.thibaudon@wanadoo.f	16
Arturo H.	Arino	Department of Environmental Biology, Biodiversity Data Analytics and Environmental Quality Group (BEQ) and Institute of Biodiversity, University of Navarra, Spain	Spain	artarip@unav.es	17
Jordina	Belmonte	Institut de Ciència i Tecnologia Ambientals (ICTA-UAB) and Department of Animal Biology, Plant Biology and Ecology, Universitat Autònoma de Barcelona, Spain	Spain	Jordina.Belmonte@uab.cat	18
Patricia	Cervigon Morales	Red Palinocam, Consejería Sanidad, Comunidad de Madrid, Madrid, Spain	Spain	patcervi@ucm.es	19
Concepción	De Linares	Institut de Ciència i Tecnologia Ambientals (ICTA-UAB) and Department of Animal Biology, Plant Biology and Ecology, Universitat Autònoma de Barcelona, Spain	Spain	concepcion.delinares@uab.cat	20
Delia	Fernández	1) Department of Biodiversity and Environmental Management, University of León, Spain, 2) Institute of Atmospheric Sciences and Climate-CNR, Bologna, Italy	Spain	mdferg@unileon.es	21
Santiago	Fernández-Rodriguez	Department of Construction, School of Technology, University of Extremadura, Avda. de la Universidad s/n, Cáceres, Spain	Spain	santiferro@unex.es	22
Antonia	Gabaldón Arguisuelas	Red Palinocam. Laboratorio Municipal de Las Rozas, Madrid, Spain	Spain	tonigabaldon@gmail.com	23
Carmen	Galán	Andalusian Institute for Earth System Research IISTA, International Campus of Excellence on Agreefood ceiA3, University of Córdoba, Spain	Spain	bv1gasoc@uco.es	24
Mónica	González-Alonso	Department of Environmental Biology, Biodiversity Data Analytics and Environmental Quality Group (BEQ) and Institute of Biodiversity, University of Navarra, Spain	Spain	mgonzalez.23@alumni.unav.es	25
Beatriz	Lara	Institute of Environmental Sciences, University of Castilla-La Mancha, Toledo, Spain	Spain	beatriz.lara@uclm.es	26
José María	Moreno Grau	Department of Chemical and Environmental Engineering, Technical University of Cartagena, Cartagena, Spain	Spain	sele.moreno@upct.es	27
José	Oteros	Andalusian Institute for Earth System Research IISTA, International Campus of Excellence on Agreefood ceiA3, University of Córdoba, Spain	Spain	oterosjose@gmail.com	28
Rosa	Pérez-Badia	Institute of Environmental Sciences, University of Castilla-La Mancha, Toledo, Spain	Spain	Rosa.Perez@uclm.es	29
Anabel	Pérez-de-Zabalza	Department of Environmental Biology, Biodiversity Data Analytics and Environmental Quality Group (BEQ) and Institute of Biodiversity, University of Navarra, Spain	Spain	aperezdezab@unav.es	30
Antonio	Picornell	Department of Botany and Plant Physiology, University of Malaga, Malaga, Spain	Spain	picornell@uma.es	31
Marta	Recio	Department of Botany and Plant Physiology, University of Malaga, Malaga, Spain	Spain	martarc@uma.es	32

First Name	Last Name	Affiliation	Country	Email	ORDER
Estrella	Robles	Department of Environmental Biology, Biodiversity Data Analytics and Environmental Quality Group (BEQ) and Institute of Biodiversity, University of Navarra, Spain	Spain	erobles@unav.es	33
Alberto	Rodríguez-Fernández	Department of Biodiversity and Environmental Management, University of León, Spain	Spain	arodrf@unileon.es	34
F. Javier	Rodríguez-Rajo	CITACA, Sciences Faculty, University of Vigo, Ourense, Spain	Spain	javirajo@uvigo.es	35
Jesús	Rojo	Institute of Environmental Sciences, University of Castilla-La Mancha, Toledo, Spain	Spain	jesus.rojo@uclm.es	36
Luis	Ruiz Valenzuela	Department Animal Biology, Plant Biology and Ecology, University of Jaén, Spain	Spain	lvalenzu@ujaen.es	37
Karl-Christian	Bergmann	German Pollen Information Service Foundation, Berlin and Charité – Universitätsmedizin Berlin, corporate member of Freie Universität Berlin, Humboldt-Universität zu Berlin, and Berlin Institute of Health, Berlin, Germany	Germany	karlchristianbergmann@gmail.com	38
Barbora	Werchan	German Pollen Information Service Foundation, Berlin and Charité – Universitätsmedizin Berlin, corporate member of Freie Universität Berlin, Humboldt-Universität zu Berlin, and Berlin Institute of Health, Berlin, Germany	Germany	barbora.werchan@charite.de	39
Matthias	Werchan	German Pollen Information Service Foundation, Berlin and Charité – Universitätsmedizin Berlin, corporate member of Freie Universität Berlin, Humboldt-Universität zu Berlin, and Berlin Institute of Health, Berlin, Germany	Germany	matthias.werchan@charite.de	40
Jeroen T. M.	Buters	Center of Allergy & Environment (ZAUM), Member of the German Center for Lung Research (DZL), Technische Universität München/Helmholtz Center, Munich, Germany	Germany	buters@tum.de	41
Maximilian	Bastl	Aerobiology and Pollen Information Research Unit, Department of Oto-Rhino-Laryngology, Medical University of Vienna, Austria	Austria	maximilian.bastl@meduniwien.ac.at	42
Susanne	Dunker	Department of Physiological Diversity, Helmholtz Centre for Environmental Research GmbH – UFZ, German Centre for integrative Biodiversity Research - iDiv, Leipzig, Germany	Germany	susanne.dunker@ufz.de	43
Thomas	Hornick	Department of Physiological Diversity, German Centre for integrative Biodiversity Research - iDiv, Helmholtz Centre for Environmental Research GmbH – UFZ, Leipzig, Germany	Germany	thomas.hornick@ufz.de	44
Nestor	González Roldán	1) Group of Allergobiochemistry, Research Center Borstel, Leibniz Lung Center, Airway Research Center North (ARCN), German Center for Lung Research (DZL), Borstel, Germany, 2) German Pollen Information Service Foundation, Berlin, Germany	Germany	ngonzalez@fz-borstel.de	45
Stefan	Gilge	Research Center Human Biometeorology, German Meteorological Service, Freiburg, Germany	Germany	Stefan.Gilge@dwd.de	46
Bernard	Clot	Federal Office of Meteorology and Climatology MeteoSwiss, Payerne, Switzerland	Switzerland	bernard.clot@meteoswiss.ch	47
Stanley	Finemann	Department of Pediatrics, Emory University School of Medicine, Atlanta Allergy & Asthma, Marietta, Atlanta, Georgia, USA	USA	sfineman@atlantaallergy.com	48
Linda	Ford	The Asthma & Allergy Center, Bellevue, Nebraska, USA	USA	lford@asthmaandallergycenter.com	49
Robert Anthony	Gomez	Allergy Research Laboratory, Lackland, San Antonio, Texas, USA	USA	robert.a.gomez38.civ@mail.mil	50

First Name	Last Name	Affiliation	Country	Email	ORDER
Sanjay	Kamboj	LSU Healthcare Network Clinics, New Orleans, Louisiana, USA	USA	skambo@lsuhsc.edu	51
Wayne	Wilhelm	Environmental Health Laboratories, Saint Louis County Department of Public Health, Berkeley, Missouri, USA	USA	wwilhelm@stlouisco.com	52
Paul J.	Beggs	Department of Earth and Environmental Sciences, Faculty of Science and Engineering, Macquarie University, Sydney, Australia	Australia	paul.beggs@mq.edu.au	53
Pamela	Burton	Department of Medicine, Campbelltown Hospital, Campbelltown, Sydney, Australia	Australia	Pamela.Burton@health.nsw.gov.au	54
Janet M.	Davies	1) School of Biological and Environmental Science, Centre Immunity and Infection Control and Centre for Environment, Institute of Health and Biomedical Innovation, Queensland University of Technology, Brisbane, Queensland, Australia, 2) Office of Research, Metro North Hospital and Health Service, Herston, Queensland, Australia	Australia	j36.davies@qut.edu.au	55
Simon Graeme	Haberle	Archaeology and Natural History, School of Culture, History and Language, College of Asia and the Pacific, Australian National University, Canberra, Australia	Australia	simon.haberle@anu.edu.au	56
Constance Helen	Katelaris	1) Immunology and Allergy Unit, Department of Medicine, Campbelltown Hospital, Sydney, Australia, 2) Immunology and Allergy Unit, Western Sydney University, Campbelltown, Sydney, Australia	Australia	Connie.Katelaris@health.nsw.gov.au	57
Ben	Keaney	Archaeology and Natural History, School of Culture, History and Language, College of Asia and the Pacific, Australian National University, Canberra, Australia	Australia	Ben.Keaney@anu.edu.au	58
Andelija	Milic	School of Biological and Environmental Science, Centre Immunity and Infection Control and Centre for Environment, Institute of Health and Biomedical Innovation, Queensland University of Technology, Brisbane, Queensland, Australia	Australia	a.milic@qut.edu.au	59
Victoria	Miller	Archaeology and Natural History, School of Culture, History and Language, College of Asia and the Pacific, Australian National University, Canberra, Australia	Australia	victoria.miller@anu.edu.au	60
Shanice	van Haeften	School of Biological and Environmental Science, Centre Immunity and Infection Control and Centre for Environment, Institute of Health and Biomedical Innovation, Queensland University of Technology, Brisbane, Queensland, Australia	Australia	s.vanhaeften@qut.edu.au	61
Maira	Bonini	Agency for Health Protection of Metropolitan Area of Milan, Milan, Italy	Italy	Mbonini@ats-milano.it	62
Anna	Bordin	Department of Cardiologic, Thoracic & Vascular Sciences - Occupational Health – Allergology, University of Padua	Italy	anna.bordin@unipd.it	63
Valentina	Ceriotti	Agency for Health Protection of Metropolitan Area of Milan, Milan, Italy	Italy	vceriotti@ats-milano.it	64
Fabiana	Cristofolini	Research and Innovation Centre, Fondazione Edmund Mach, San Michele all'Adige, Italy	Italy	fabiana.cristofolini@fmach.it	65
Antonella	Cristofori	Research and Innovation Centre, Fondazione Edmund Mach, San Michele all'Adige, Italy	Italy	antonella.cristofori@fmach.it	66
Elena	Gottardini	Research and Innovation Centre, Fondazione Edmund Mach, San Michele all'Adige, Italy	Italy	elena.gottardini@fmach.it	67
Guido	Marcer	Department of Cardiologic, Thoracic & Vascular Sciences - Occupational Health – Allergology, University of Padua, Italy	Italy	guido.marcer@unipd.it	68
Paolo	Marraccini	Department of Occupational Medicine, Occupational Allergy Unit, Fondazione IRCCS Ca' Granda Ospedale Maggiore Policlinico, Milan, Italy	Italy	paolo.marraccini@policlinico.mi.it	69

First Name	Last Name	Affiliation	Country	Email	ORDER
Paolo	Mascagni	Clinical Unit of Occupational Health, Department of Occupational, Environmental and Community Medicine, Desio Hospital, Desio, Italy	Italy	p.mascagni@asst-monza.it	70
Antonio	Meriggi	Allergy and Immunology Unit of Pavia, IRCCS Institute, Istituti Clinici Scientifici Maugeri, Pavia,	Italy	antonio.meriggi@icsmaugeri.it	71
Loretta	Pace	Department of Life, Health and Environmental Sciences, University of L'Aquila, L'Aquila, Italy	Italy	loretta.pace@univaq.it	72
Alberto	Pini	Laboratorio di Prevenzione, ATS della Montagna, Sondrio, Italy	Italy	a.pini@ats-montagna.it	73
Maria Cristina	Tacca	Clinical Unit of Occupational Health, Department of Occupational, Environmental and Community Medicine, Desio Hospital, Desio, Italy	Italy	m.tacca@asst-monza.it	74
Nicolas	Bruffaerts	Mycology & Aerobiology service, Sciensano, Brussels, Belgium	Belgium	nicolas.bruffaerts@sciensano.be	75
Lucie	Hoebeke	Mycology & Aerobiology service, Sciensano, Brussels, Belgium	Belgium	lucie.hoebeke@sciensano.be	76
Beverley	Adams-Groom	School of Science and the Environment, University of Worcester, Worcester, United Kingdom	UK	b.adams-groom@worc.ac.uk	77
Catherine H.	Pashley	Department of Respiratory Sciences, Institute for Lung Health, University of Leicester, Leicester, UK	UK	chp5@le.ac.uk	78
Jack	Satchwell	Department of Respiratory Sciences, Institute for Lung Health, University of Leicester, Leicester, UK	UK	js660@le.ac.uk	79
Carsten	Skjøth	School of Science and the Environment, University of Worcester, Worcester, United Kingdom	UK	c.skjoth@worc.ac.uk	80
Fiona A.	Symon	Department of Respiratory Sciences, Institute for Lung Health, University of Leicester, Leicester, UK	UK	fas4@le.ac.uk	81
Celia M.	Antunes	Institute of Earth Sciences (ICT) & Department of Chemistry, School of Sciences and Technology, University of Evora, Evora, Portugal	Portugal	cmma@uevora.pt	82
Elsa	Caeiro	1) SPAIC-Sociedade Portuguesa de Alergologia e Imunologia Clínica, Lisbon, Portugal, 2) MED-Mediterranean Institute for Agriculture, Environment and Development, Institute for Advanced Studies and Research, University of Évora, Évora, Portugal	Portugal	elcaeiro@yahoo.com	83
Irene Gomes Câmara	Camacho	Faculty of Life Sciences, Madeira University, Funchal, Portugal	Portugal	ireneg@staff.uma.pt	84
Ana R.	Costa	Institute of Earth Sciences (ICT) & Department of Chemistry, School of Sciences and Technology, University of Evora, Evora, Portugal	Portugal	acrc@uevora.pt	85
Ricardo João Ratola Capela	Deus	Instituto Português do Mar e da Atmosfera (IPMA), Lisbon	Portugal	ricardo.deus@ipma.pt	86
Manuel Branco	Ferreira	1) Clinica Universitária Imunoalergologia - Faculdade de Medicina Universidade Lisboa, 2) Serviço de Imunoalergologia - Centro Hospitalar Universitário Lisboa Norte, 3) SPAIC-Sociedade Portuguesa de Alergologia e Imunologia Clínica	Portugal	mbrancoferreira@gmail.com	87
Joao Almeida Lopes	Fonseca	CINTESIS, Center for Health Technology and Services, Research & MEDCIDS, Department of Community Medicine, Health Information and Decision Sciences, Faculty of Medicine, University of Porto, Portugal	Portugal	fonseca.ja@gmail.com	88
Ana	Galveias	Institute of Earth Sciences (ICT) & Department of Chemistry, School of Sciences and Technology, University of Evora, Evora, Portugal	Portugal	anagalveias@gmail.com	89
Helena	Ribeiro	Department of Geosciences, Environment and Spatial Plannings, Faculty of Sciences, University of Porto, Porto, Portugal	Portugal	helena.ribeiro@fc.up.pt	90

First Name	Last Name	Affiliation	Country	Email	ORDER
Beatriz	Tavares	1) Centro Hospitalar e Universitário de Coimbra, Coimbra, Portugal, 2) SPAIC-Sociedade Portuguesa de Alergologia e Imunologia Clínica	Portugal	beatriztavares@net.sapo.pt	91
Łukasz	Grewling	Laboratory of Aerobiology, Department of Systematic and Environmental Botany, Faculty of Biology, Adam Mickiewicz University in Poznań, Poznań, Poland	Poland	grewling@amu.edu.pl	92
Agnieszka	Grinn-Gofroń	Institute of Biology, University of Szczecin, Szczecin, Poland	Poland	agnieszka.grinn-gofron@usz.edu.pl	93
Dariusz	Jurkiewicz	Department of Otolaryngology with Division of Cranio-Maxillo-Facial Surgery, Military Institute of Medicine, Warsaw, Poland	Poland	djurkiewicz@lekarz.net	94
Ewa	Kalinowska	Allergen Research Center, Warsaw, Poland	Poland	ekalinowska@obas.pl	95
Agnieszka	Lipiec	Department of the Prevention of Environmental Hazards and Allergology, Medical University of Warsaw, Warsaw, Poland	Poland	alipiec@wum.edu.pl	96
Dorota	Myszkowska	Department of Clinical and Environmental Allergology, Jagiellonian University Medical College, Krakow, Poland	Poland	dorota.myszkowska@uj.edu.pl	97
Krystyna	Piotrowska-Weryszko	Department of Botany and Plant Physiology, University of Life Sciences in Lublin, Lublin, Poland	Poland	krystyna.piotrowska@up.lublin.pl	98
Malgorzata	Puc	Institute of Marine & Environmental Sciences, University of Szczecin, Poland	Poland	malgorzata.puc@usz.edu.pl	99
Anna	Rapiejko	Allergen Research Center, Warsaw, Poland	Poland	anna.rapiejko@obas.pl	100
Piotr	Rapiejko	1) Allergen Research Center, Warsaw, Poland, 2) Department of Otolaryngology with Division of Cranio-Maxillo-Facial Surgery, Military Institute of Medicine, Warsaw, Poland	Poland	piotr@rapiejko.pl	101
Elżbieta	Weryszko-Chmielewska	Department of Botany and Plant Physiology, University of Life Sciences in Lublin, Lublin, Poland	Poland	elaweryszko@wp.pl	102
Monika	Ziemianin	Department of Clinical and Environmental Allergology, Jagiellonian University Medical College, Krakow, Poland	Poland	monika.wandas@uj.edu.pl	103
Dilys	Berman	Allergy Immunology Unit UCT Lung Institute George Street Mowbray Cape Town South Africa	South Africa	Dilys.Berman@uct.ac.za	104
Werner	Hoek	Gariep Medi Clinic Kimberley and Robert Mangaliso Sebokwe Hospital (Previous: Kimberley Hospital Complex), University of the Free State, South Africa	South Africa	drhoek@surgeon.co.za	105
Ahmed Ismail	Manjra	Hiway Medical Centre, Westville Hospital, Durban, South Africa	South Africa	manjra@mweb.co.za	106
Jonathan	Peter	Allergology and Clinical Immunology, Department of Medicine, Groote Schuur Hospital, University of Cape Town Lung Institute, Groote Schuur, South Africa	South Africa	Jonny.Peter@uct.ac.za	107
Åslög	Dahl	Department of Biological and Environmental Sciences, University of Gothenburg, Gothenburg, Sweden	Sweden	aslog.dahl@bioenv.gu.se	108
Agneta	Ekebom	Palynological Laboratory, Environmental research and monitoring, Swedish Museum of Natural History, Stockholm, Sweden	Sweden	agneta.ekebom@nrm.se	109
Barbara	Stjepanovic	Andrija Stampar Teaching Institute of Public Health, Zagreb, Croatia	Croatia	barbara.stjepanovic@stampar.hr	110
Ana	Večenaj	Andrija Stampar Teaching Institute of Public Health, Zagreb, Croatia	Croatia	ana.vecenaj@stampar.hr	111
Sevcan	Celenk	Bursa Uludag University, Arts and Science Faculty, Biology Department, Aerobiology Laboratuary, Görükle-Bursa, Turkey	Turkey	sevcancelenk@hotmail.com	112

First Name	Last Name	Affiliation	Country	Email	ORDER
Özlem	Göksel	Aerobiology Team, Translational Pulmonology Research Group (EgeTPAG), EgeSAM Ege University Lung Research Center, Izmir, Turkey	Turkey	goksel.ozlem@gmail.com	113
Tuncay	Göksel	Aerobiology Team, Translational Pulmonology Research Group (EgeTPAG), EgeSAM Ege University Lung Research Center, Izmir, Turkey	Turkey	tuncaygoksel@gmail.com	114
Aykut	Guvensen A	Aerobiology Team, Translational Pulmonology Research Group (EgeTPAG), EgeSAM Ege University Lung Research Center, Izmir, Turkey,	Turkey	aykut.guvensen@ege.edu.tr	115
Nur Munevver	Pinar	Department of Biology, Faculty of Science, Ankara University, Ankara, Turkey	Turkey	pinar@science.ankara.edu.tr	116
Cansin	Sackesen	Division of Pediatric Allergy, School of Medicine, Koc University, Istanbul, Turkey	Turkey	csackesen@ku.edu.tr	117
Aydar	Acar Sahin	Department of Biology, Faculty of Science, Ankara University, Ankara, Turkey	Turkey	aydanacar24@gmail.com	118
Ulas	Uguz U	Aerobiology Team, Translational Pulmonology Research Group (EgeTPAG), EgeSAM Ege University Lung Research Center, Izmir, Turkey	Turkey	ulasoguz@gmail.com	119
Duygu	Yazici	Cellular and Molecular Medicine, KUTTAM, Graduate School of Health Sciences, Koc University, Istanbul, Turkey	Turkey	dyazici17@ku.edu.tr	120
Dóra	Kajtor-Apatini	National Public Health Center, Budapest, Hungary	Hungary	apatini.dora@nnk.gov.hu	121
Donát	Magyar	National Public Health Center, Budapest, Hungary	Hungary	magyar.donat@gmail.com	122
Tamás	Szigeti	National Public Health Center, Budapest, Hungary	Hungary	szigeti.tamas@nnk.gov.hu	123
Branko	Sikoparija	BioSense Institute - Research Institute for Information Technologies in Biosystems, University of Novi Sad, Novi Sad, Serbia	Serbia	sikoparijabranko@biosense.rs	124
Andreja	Kofol Seliger	National Laboratory of Health, Environment and Food, Ljubljana, Slovenia	Slovenia	andreja.kofol.seliger@nlzoh.si	125
Anja	Simčič	National Laboratory of Health, Environment and Food, Ljubljana, Slovenia	Slovenia	anja.simcic@nlzoh.si	126
Jae	Oh	Department of Pediatrics Hanyang University Guri Hospital, Department of Pediatrics, College of Medicine, Hanyang University, Seoul, South Korea	South Korea	jaewonoh@hanyang.ac.kr	127
Athanasios	Charalampopoulos	Department of Ecology, School of Biology, Faculty of Sciences, Aristotle University of Thessaloniki, Thessaloniki, Greece	Greece	athchara@bio.auth.gr	128
Despoina	Vokou	Department of Ecology, School of Biology, Faculty of Sciences, Aristotle University of Thessaloniki, Thessaloniki, Greece	Greece	vokou@bio.auth.gr	129
Karen	Rasmussen	Astma-Allergi Danmark, Roskilde, Denmark	Denmark	kr@astma-allergi.dk	130
Laura Beatriz	Barrionuevo	Instituto de Alergia e Inmunologia del Sur, Bahia Blanca, Argentina	Argentina	laurabbarrionuevo@gmail.com	131
German Dario	Ramon	Instituto de Alergia e Inmunologia del Sur, Bahia Blanca, Argentina	Argentina	germanramon2004@hotmail.com	132
Letty A.	de Weger	Department of Pulmonology, Leiden University Medical Center, Leiden, The Netherlands	Netherlands	l.a.de_weger@lumc.nl	133
Mieke M.J.F.	Koenders	Algemeen Klinisch Laboratorium, Elkerliek ziekenhuis Helmond, The Netherlands	Netherlands	mkoenders@elkerliek.nl	134
Arnold J.H.	van Vliet	Environmental Systems Analysis Group, Wageningen University	Netherlands	arnold.vanvliet@wur.nl	135
Jozef	Dušička	Department of Botany, Faculty of Natural Sciences, Comenius University in Bratislava, Bratislava,	Slovakia	jozef.dusicka@uniba.sk	136

First Name	Last Name	Affiliation	Country	Email	ORDER
Janka	Lafférsová	Department of Medical Microbiology, Section of Microbiology and Enviromental Biology, Regional Authority of Public Health Banska Bystrica, Slovakia	Slovakia	janka.laffersova@gmail.com	137
Jana	Ščevková	Department of Botany, Faculty of Natural Sciences, Comenius University in Bratislava, Bratislava,	Slovakia	jana.scevkova@uniba.sk	138
Ondřej	Rybníček	Paediatric Department, Allergy Unit, Masaryk University and University Hospital Brno, Brno, Czech Republic	Czech Republic	rybnicek.o@seznam.cz	139
Frances	Coates	Aerobiology Research Laboratory, Ottawa, Canada	Canada	frances@aerobiology.ca	140
Dawn	Jurgens	Aerobiology Research Laboratory, Ottawa, Canada	Canada	dawn@aerobiology.ca	141
Ingrida	Šaulienė	Department of Engineering, Siauliai University, Siauliai, Lithuania	Lithuania	ingrida.sauliene@su.lt	142
Elena	Severova	Moscow State University, Biological Faculty, Moscow, Russia	Russia	elena.severova@mail.ru	143
Victoria	Rodinkova	Laboratory of the Environmental Factors Investigation, Pharmacy Department, National Pirogov Memorial Medical University, Vinnytsia, Ukraine	Ukraine	vikarodi@gmail.com	144
Mykyta	Bortnyk	1) Laboratory of the Environmental Factors Investigation, Pharmacy Department, National Pirogov Memorial Medical University, Vinnytsia, Ukraine, 2) Vasyl' Stus Donetsk National University, Vinnytsia, Ukraine	Ukraine	m.bortnyk@donnu.edu.ua	145
Olena	Palamarchuk	Laboratory of the Environmental Factors Investigation, Pharmacy Department, National Pirogov Memorial Medical University, Vinnytsia, Ukraine	Ukraine	olenavolk80@gmail.com	146
Maryna	Yasniuk	Laboratory of the Environmental Factors Investigation, Pharmacy Department, National Pirogov Memorial Medical University, Vinnytsia, Ukraine	Ukraine	yasnyukmarina@gmail.com	147
Maria	Louna-Korteniemi	Biodiversity Unit, University of Turku, Finland	Finland	amloun@utu.fi	148
Sanna	Pätsi	Biodiversity Unit, University of Turku, Finland	Finland	smpats@utu.fi	149
Annika	Saarto	Biodiversity Unit, University of Turku, Finland	Finland	annika.saarto@utu.fi	150
Linnea	Toiviainen	Biodiversity Unit, University of Turku, Finland	Finland	liesto@utu.fi	151
Olga	Sozinova	Faculty of Geography and Earth Sciences, University of Latvia, Riga, Latvia	Latvia	olga.sozinova@lu.lv	152
Peng	Jia	1) Department of Land Surveying and Geo-Informatics, The Hong Kong Polytechnic University, Hong Kong, China, 2) International Institute of Spatial Lifecourse Epidemiology (ISLE), Hong Kong, China	China	jiapengff@hotmail.com	153
